# Neuronophagia and microglial nodules in a SARS-CoV-2 patient with cerebellar hemorrhage

**DOI:** 10.1186/s40478-020-01024-2

**Published:** 2020-08-26

**Authors:** Osama Al-Dalahmah, Kiran T. Thakur, Anna S. Nordvig, Morgan L. Prust, William Roth, Angela Lignelli, Anne-Catrin Uhlemann, Emily Happy Miller, Shajo Kunnath-Velayudhan, Armando Del Portillo, Yang Liu, Gunnar Hargus, Andrew F. Teich, Richard A. Hickman, Kurenai Tanji, James E. Goldman, Phyllis L. Faust, Peter Canoll

**Affiliations:** 1 Department of Pathology and Cell Biology, Vagelos College of Physicians and Surgeons, Columbia University Irving Medical Center and the New York Presbyterian Hospital, New York, NY USA; 2 Department of Neurology, Vagelos College of Physicians and Surgeons, Columbia University Irving Medical Center and the New York Presbyterian Hospital, New York, NY USA; 3 Department of Radiology, Vagelos College of Physicians and Surgeons, Columbia University Irving Medical Center and the New York Presbyterian Hospital, New York, NY USA; 4 Division of Infectious Diseases, Department of Medicine, Vagelos College of Physicians and Surgeons, Columbia University Irving Medical Center and the New York Presbyterian Hospital, New York, NY USA

**Keywords:** Microglial nodules, Neuronophagia, SARS-CoV-2, COVID-19, Neuropathology

## Abstract

We document the neuropathologic findings of a 73-year old man who died from acute cerebellar hemorrhage in the context of relatively mild SARS-CoV2 infection. The patient developed sudden onset of headache, nausea, and vomiting, immediately followed by loss of consciousness on the day of admission. Emergency medical services found him severely hypoxemic at home, and the patient suffered a cardiac arrest during transport to the emergency department. The emergency team achieved return of spontaneous circulation after over 17 min of resuscitation. A chest radiograph revealed hazy bilateral opacities; and real-time-PCR for SARS-CoV-2 on the nasopharyngeal swab was positive. Computed tomography of the head showed a large right cerebellar hemorrhage, with tonsillar herniation and intraventricular hemorrhage. One day after presentation, he was transitioned to comfort care and died shortly after palliative extubation. Autopsy performed 3 h after death showed cerebellar hemorrhage and acute infarcts in the dorsal pons and medulla. Remarkably, there were microglial nodules and neuronophagia bilaterally in the inferior olives and multifocally in the cerebellar dentate nuclei. This constellation of findings has not been reported thus far in the context of SARS-CoV-2 infection.

## Introduction

Symptomatic SARS-CoV-2 infection presents as a respiratory syndrome with upper and lower respiratory systems manifestations, characterized by cough, dyspnea, fever, chills, hyposmia, and ageusia [5, 20]. Male patients [9] as well as patients with comorbidities such as diabetes, obesity, hypertension, cardiac disease, pulmonary disease, and other chronic diseases [32], are prone to more severe manifestations. While the majority of infections are mild, severe infections can lead to serious end-organ damage due to respiratory failure, acute kidney injury, disseminated intravascular coagulation-like systemic coagulopathy [12], and thrombosis [16]. Neurologic sequelae have been reported in a number of SARS-CoV-2 patients including ischemic stroke, seizures, Guillain–Barre Syndrome, and acute necrotizing hemorrhagic leukoencephalopathy [3, 8, 14, 15, 19, 25, 35]. It is yet to be determined whether CNS manifestations are directly attributed to CNS infection by SARS-CoV-2, secondary to systemic effects in the context of hypoxia and multiorgan damage, or both.

Pathologic findings in the CNS from autopsy studies of SARS-CoV-2 patients are scarce. Most studies thus far lacked neuropathologic characterization altogether [1, 2, 30, 31, 34]. A case series describing 10 autopsies of SARS-CoV-2 decedents found no signs of encephalitis or vasculitis [22]. Another study of autopsy findings of four brains showed mild hypoxemic changes in three out of the four brains examined, but no encephalitis [21]. A recent case report of neuropathologic findings in one case of SARS-CoV-2 documented a picture of multifocal white matter hemorrhages, axonal damage in a variable perivenular distribution, and no significant perivascular inflammation. While a cortical organizing infarct was noted, neuronal necrosis, neuronophagia, microglial nodules, and vascular necrosis were not identified [24]. Most recently, a case series of 18 SARS-CoV-2 autopsies documented the presence of hypoxemic changes, perivascular inflammation in a subset of cases, and a single microglial nodule, however, the authors concluded there was no evidence of encephalitis or vasculitis. Real-time PCR identified viral transcripts in the frontal lobe, medulla and olfactory nerves in the majority of cases [29].

In this report, we describe the neuropathologic findings from a confirmed SARS-CoV-2 autopsy, where we describe a case of marked neuronophagia involving the inferior olives and dentate nuclei.

## Case report

**Fig. 1:**
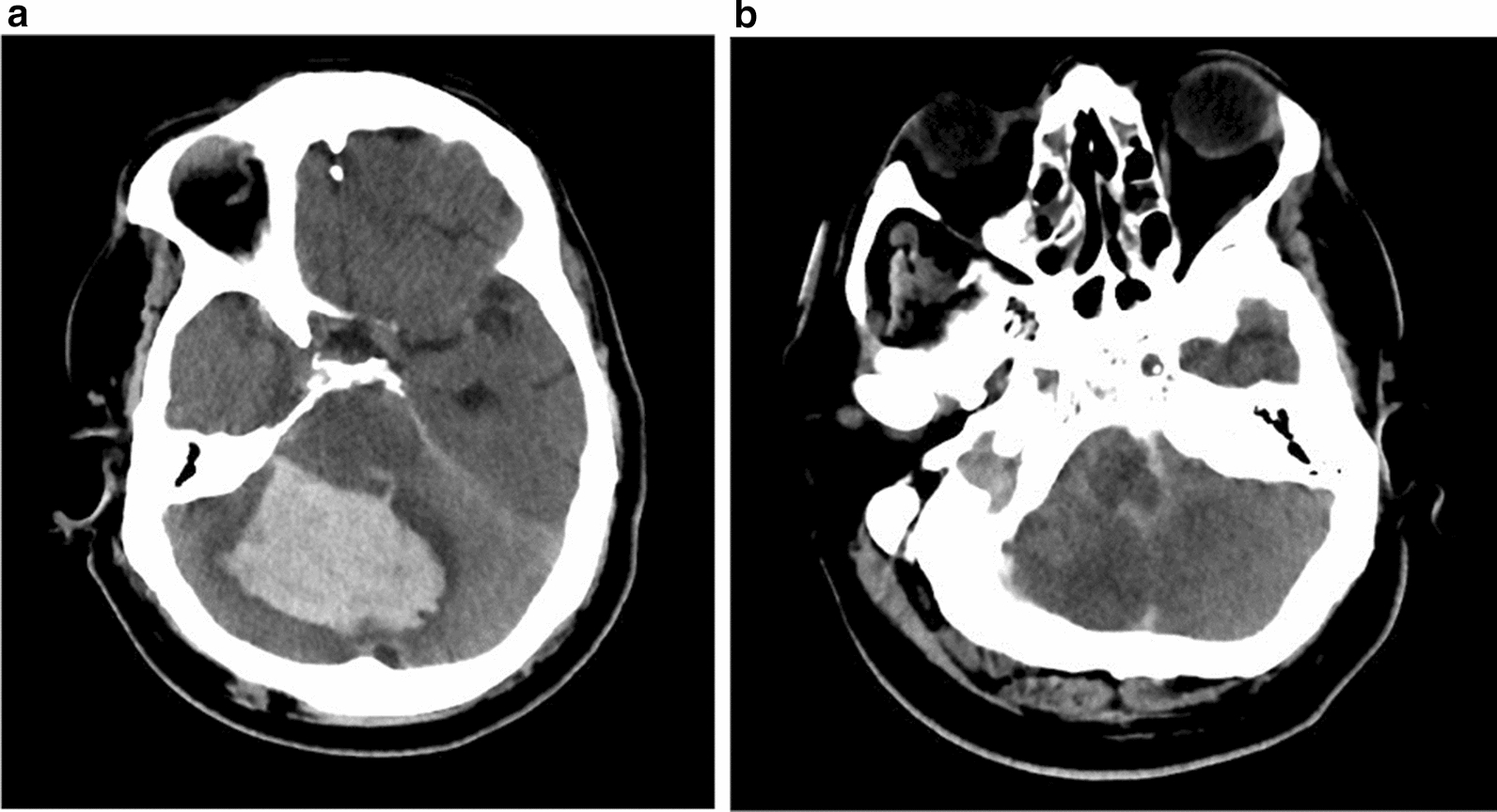
Axial non-contrasted head CT scans of the posterior fossa. The images show a large cerebellar hematoma with surrounding edema (**a**) as well as edema in the medulla (**b**)

A 73-year-old man with hypertension and type II diabetes mellitus presented with sudden onset of headache, followed by nausea, vomiting, and loss of consciousness. He was fully functional at baseline and had no recent infectious symptoms prior to presentation. On the day of admission, he complained to his caretaker of a sudden severe headache while eating lunch. When a second caregiver arrived, he had become unarousable. Emergency medical services were called, and his initial vital signs revealed an oxygen saturation of 30%. He was intubated and oxygen saturation of 80% was achieved with bag and valve ventilation. During transportation to the emergency department, he went into cardiac arrest with pulseless electrical activity. During the resuscitation, out of suspicion for esophageal placement of the endotracheal tube, he was re-intubated. Return of spontaneous circulation was achieved after at least 17 min. His initial vital signs were notable for temperature 36.2 °C, heart rate 113 beats per minute, blood pressure 113/72 mmHg, and oxygen saturation 100% on mechanical ventilation. Chest radiograph showed hazy bilateral opacities, and nasopharyngeal swab for SARS-CoV-2 RNA was positive. Laboratory studies were notable for WBC 13.3 × 10^9^/L, hemoglobin 14.6 g/dL, platelets 346 × 10^9^/L, serum creatinine 1.16 mg/dL, and serum lactate 12.0. C-reactive protein (CRP) was elevated 8.7 mg/L (normal is less than 3 mg/L), and erythrocyte sedimentation rate (ESR) was 27 mm (normal is 0–10 mm). No other inflammatory studies were performed. Liver chemistries and coagulation studies were within normal limits. Computed tomography (CT) of the head showed a 6 × 4 cm right cerebellar intra-parenchymal hemorrhage (Fig. 1a), edema and compression of the medulla (Fig. 1b), and tonsillar herniation, The fourth ventricle was compressed and there was secondary obstructive hydrocephalus as well as intraventricular hemorrhage. CT angiography of the head did not reveal an underlying vascular lesion. With no improvement after 18 h, his care was transitioned to comfort measures and he died within minutes of palliative extubation.

## Neuropathologic findings

### Gross findings

The fixed brain weighed 1350 g. The cerebral hemispheres were symmetrical and edematous with flattening of the gyri and thinning of the sulci. No cingulate or uncal herniations were recognized. However, upward herniation of the midbrain was noted. The leptomeninges over the base of the brain showed subarachnoid hemorrhage. The vessels at the base of the brain showed a normal configuration of the Circle of Willis and atherosclerosis involving the basilar artery (80% stenosis), and the remaining vessels (30–40% stenosis). No aneurysms were identified. The left vertebral artery was smaller than the right. The cerebellar hemispheres were edematous, dusky, softened, and disrupted on the right side. There was a 6.0 × 5.0 × 4.0 cm hematoma embedded within the right deep cerebellar white matter centered at the level of the middle cerebellar peduncle. Bilateral tonsillar herniation as well as subarachnoid hemorrhages in the ventral pontine region and in the midline dorsal cerebellum were documented. Sequential sections through the supra-tentorial tissues showed intra-ventricular hemorrhage and marked dilatation of both the lateral and third ventricles. No ventricular displacement was noted. The cortex was apparently normal, and notably the subcortical white matter did not reveal any gross hemorrhages. The aqueduct of Sylvius was filled with hemorrhage. Sequential sections of the midbrain, pons, and medulla at right angles to the neuraxis showed no Duret hemorrhages. All brain stem structures were distorted and dusky. The medulla and pons were shifted across the midline to the left. Axial sections of the cerebellum showed dusky cerebellar cortex and hemorrhage in the right deep cerebellar white matter at the level of the middle cerebellar peduncle.

### Microscopic findings

Histologic examination of the Hematoxylin and Eosin (H&E) stained brain sections showed severe global hypoxic changes with scattered hypereosinophilic shrunken neurons in the cerebral cortex, striatum, thalamus, amygdala, hippocampus, midbrain, pontine nuclei, medullary nuclei, and Purkinje cell layer. There were acute infarcts in the dorsal medulla and pontine tectum, likely attributable to herniation. The cerebellar white matter showed extravasation of red blood cells and neutrophilic infiltration of the parenchyma, consistent with early acute infarction (not shown). There was no evidence of vasculitis.

**Fig. 2:**
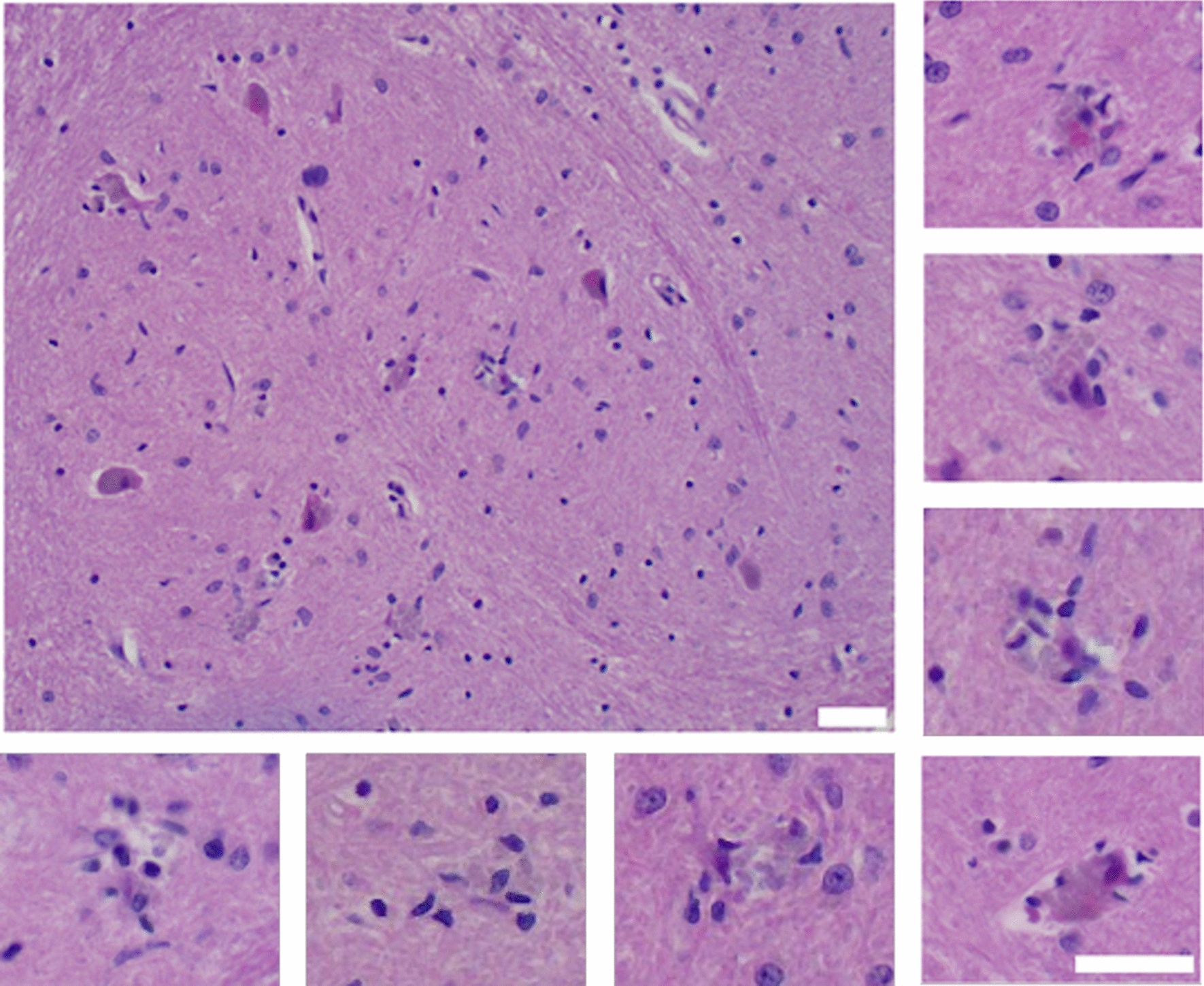
Microglial nodules in the inferior olivary nuclei. Representative images of the inferior olivary nuclei showing microglial nodules and neuronophagia. The large panel (top left) shows an overall view of the left inferior olivary nucleus, with microglial nodules and neuronal loss. Several olivary neurons appear hyper-eosinophilic. The insets show higher power images of individual examples of microglial nodules with varying degrees of neuronal damage. Scale bars measure 100 µm

**Fig. 3:**
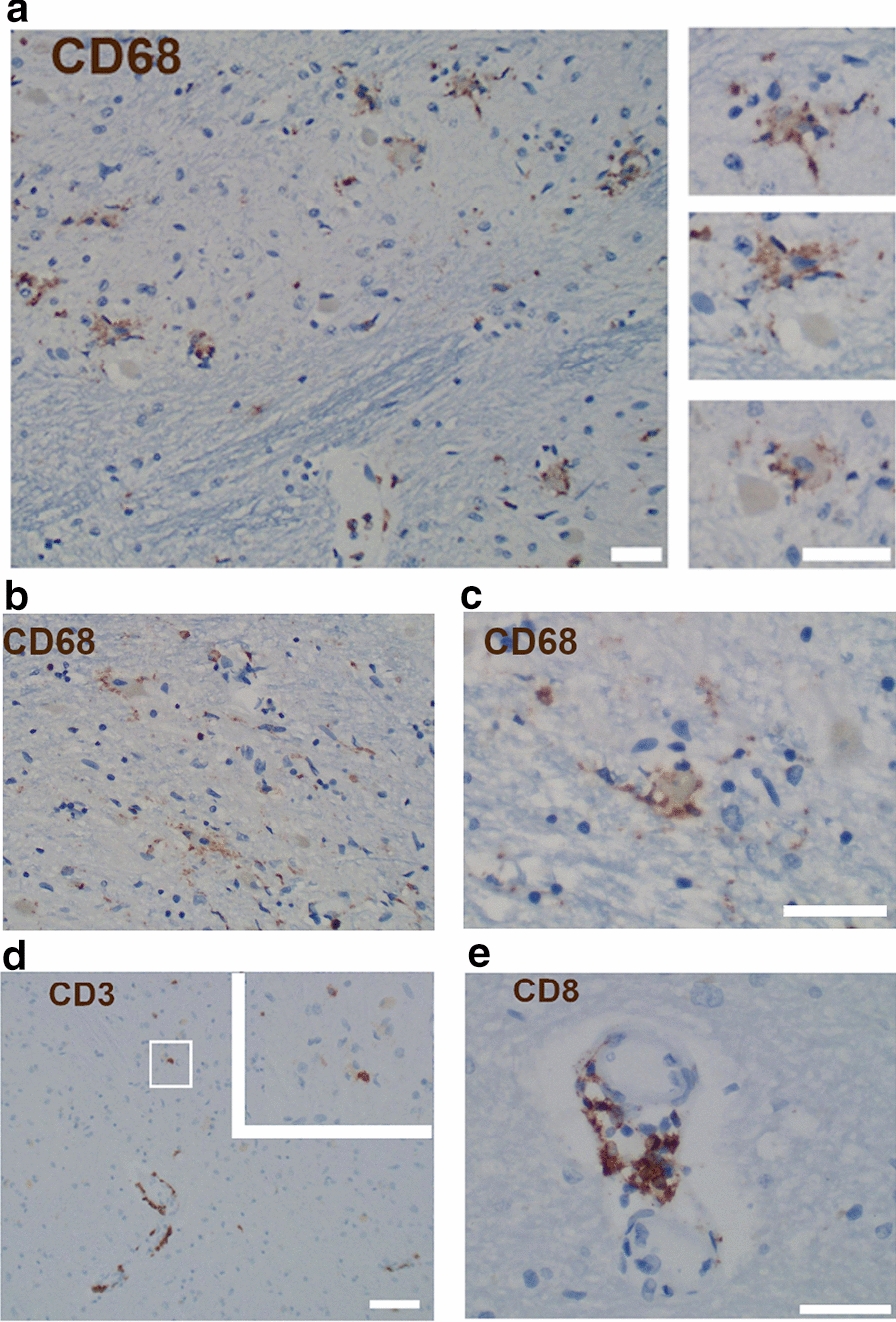
Neuronophagia in the inferior olivary and the dentate nuclei. **a** Neuronophagia highlighted by a CD68 immunohistochemical stain. Note the clusters of activated microglia surrounding shrunken and apparently normal inferior olivary neurons. Higher magnification images are shown on the right. **b**, **c** Immunohistochemical stain for CD68 showing microglial activation and neuronophagia in the cerebellar dentate nucleus. **d** Immunohistochemical stain for CD3 highlighted perivascular lymphocytes around venules in the medulla and sparse parenchymal infiltrates. The inset shows an example of a T cell juxtaposed to an olivary neuron. **e** Immunohistochemical stain for CD8 in the cerebellar white matter shows a subset of perivascular lymphocytes were CD8 positive. No CD8 lymphocytes were seen in the parenchyma. Scale bars measure 100 µm

The inferior olives showed bilateral, multifocal, numerous microglial nodules (Fig. 2), and neuronophagia, which is highlighted by a CD68 immunostain (KP1 clone) (Fig. 3a). Microglial activation and neuronophagia were also seen in the cerebellar dentate nuclei, as highlighted by CD68 immunostains (Fig. 3b, c). Small collections of perivascular lymphocytes were identified around medullary venules (Fig. 3d) as highlighted by a CD3 immunostain. Only sparse parenchymal lymphocytes were seen next to olivary neurons (Fig. 3d). CD8 immunostain showed that a subset of perivascular lymphocytes was cytotoxic (Fig. 3e). No CD8 cells were identified in microglial nodules or near olivary neurons. We also identified mild perivascular inflammatory infiltrates in multiple regions including the corpus callosum, corpus striatum, thalamus, hippocampus, midbrain, and pons. An acute infarct was identified in the pontine tectum. Notably, we did not identify microglial nodules or neuronophagia in other cortical or subcortical structures. There was white matter edema, but no loss of myelin. Astrogliosis was noted in the superior frontal and orbital cortices on GFAP immunostain. Microglial activation in the cortex was not evident on CD68 immunostains. Sparse leptomeningeal lymphocytes were identified in multiple regions focally. Immunohistochemical staining of viral particles was negative in the inferior olives and dentate nuclei using a monoclonal antibody against the SARS-CoV-2 nucleocapsid protein (40143-R001—Sino Biological©). Furthermore, in situ hybridization for SARS-CoV-2 viral RNA was negative in the medulla and cerebellum. All immunohistochemical studies and in situ hybridization were conducted in the Department of Pathology immunohistochemistry core using routine protocols with the Leica™ Bond auto-stainer. All antibodies and assays used are available for clinical diagnostics.

Other relevant findings included expansion of perivascular spaces in the ventral thalamus, sparse perivascular hemosiderin-laden macrophages around arterioles that showed mild medial thickening. There were scattered focal perivascular hemorrhages in the corpus callosum (not shown). No amyloid angiopathy was identified on Beta-Amyloid immunostains. The basilar artery showed moderate non-occlusive atherosclerosis. Ferro-calcific sclerosis was noted in the pallidal vessels. Notably, examination of the cerebellar white matter showed astrogliosis including Alzheimer’s type-2 astrocytes (not shown). The choroid plexus and pituitary gland were unremarkable. The olfactory epithelium showed chronic active inflammation. The olfactory bulb showed scattered red neurons and the tract showed no diagnostic abnormalities.

Results of real-time PCR for SARS-CoV-2 conducted as outlined in the CDC guidelines [7] demonstrated the presence of viral transcripts in the nasal epithelium (Mean CT 31.75—278 copies/microliter RNA) and cerebellar clot (Mean CT 33.0—559 copies/microliter RNA), low levels in the olfactory bulb (Mean CT 36.70—11 copies/microliter RNA) and cerebellum (Mean CT 37.17—8 copies/microliter RNA), and no detectable transcripts in the medulla. Note that real-time PCR for SARS-CoV-2 from the cerebellar clot sample was performed on a separate plate, thus, a separate standard curve was used to calculate viral copy number.

The general autopsy identified bronchopneumonia in the left lower lobe, with positive SARS-CoV-2 immunohistochemistry. The heart showed left ventricular hypertrophy, focal subendocardial fibrosis, but no myocarditis or ischemia. The liver showed steatohepatitis. The kidneys showed diffuse isometric tubular vacuolization, consistent with osmotic nephrosis. Hypertensive arteriosclerosis was mild. Blood and lung cultures were negative for bacteria and fungi.

## Discussion

The unique findings we describe in this report revealed marked neuronophagia and microglial nodules in the inferior olives and to a lesser extent the dentate nuclei, and only mild perivascular lymphocytic infiltrates. Parenchymal inflammation in the inferior olives and dentate nuclei has been described in neuroinvasive enterovirus 71 infection [33]. Radiologic evidence of encephalitis was described in a recent report of SARS-CoV-2 [26], however, the lack of prominent inflammation in our case makes encephalitis less likely. Moreover, microglial nodules with white matter perivascular hemorrhages were described by Hart and Earle [13] and Russell [27] and designated as “perivenous encephalitis”, which is associated with childhood mumps, measles, chickenpox, and less commonly vaccination. The inflammation in perivenous encephalitis is more pronounced than what we observed in this case, and prominent perivascular hemorrhages are lacking. The absence of prominent perivascular hemorrhage and demyelination rules out Acute Hemorrhagic Leuko-Encephalitis, which was recently inferred radiologically in a SARS-CoV-2 case [23].

We hypothesize that the marked neuronophagia in our case results from the cytokine-induced microglial activation, that primes microglia to phagocytose hypoxic neurons. SARS-CoV-2 infection is associated with increased systemic cytokine levels, including Interleukin-6 and Interferon-gamma [6, 17], which are known to activate microglia, and can up-regulate the expression of microglial receptors involved in phagocytosis [18]. Concomitantly, hypoxia can induce the expression of “eat me” signals on the surface of neurons, making hypoxic neurons more vulnerable to phagocytosis. Phagocytosis of viable or dying neurons has been described in hypoxic and inflammatory conditions [4]. In this way, we propose that the hypoxia and systemic inflammation act synergistically to induce neuronophagia. The main alternative hypothesis is the direct viral infection of neurons leading to neuronal death and neuronophagia. We find this alternative hypothesis unlikely because we did not identify viral proteins by immunohistochemistry, which detected viral proteins in the lung. Furthermore, viral RNA was extremely low in the brain and is likely explained by the presence of viral RNA in the blood within the specimens. This possibility is supported by our detection of viral RNA in the cerebellar blood clot. Moreover, viral infections such as Herpes Simplex and Cytomegalovirus are associated with a more prominent inflammatory response [28]. Therefore, a direct infection of neurons in our case is unlikely, especially that the medulla—where neuronophagia was most abundant—was devoid of viral transcripts. Another alternative hypothesis is that neuronophagia may be secondary to disruption of the olivary-cerebellar circuitry resulting in trans-synaptic degeneration of the olivary neurons. We do not favor this possibility because disruption of the Guillain–Mollaret triangle is generally associated with pseudo-hypertrophy of the inferior olivary nucleus [10]. This process does not involve neuronophagia, rather enlargement and cytoplasmic vacuolation of olivary neurons beginning three weeks after injury, followed later by astrocytic hypertrophy (gemistocytic astrocytes), and neuronal loss months later. In fact, in the acute phase (< 7 days), no histologic abnormalities except for pallor of the amiculum were described [11]. In addition, neuronophagia involving both inferior olives is not predicted from interruption of the circuitry on one side. Therefore, the clinico-pathologic findings in the case reported herein are incompatible with olivary pseudo-hypertrophy.

The cause of the cerebellar hemorrhage is most likely hypertensive vasculopathy. Perivascular hemosiderin-laden macrophages were identified in the cerebellar white matter adjacent to the bleed. It is interesting that the severity of these hypertensive changes was only mild in the white matter, thalamus, corpus striatum, and the kidney. We also cannot fully exclude SARS-CoV-2 vasculopathy as a potential contributor to the cerebellar hemorrhage. However, the vascular changes in this case are likely secondary to the herniation and subsequent acute infarcts. We did not identify definitive evidence of vasculitis or amyloid angiopathy.

Overall, the constellation of findings we describe are unique. To our knowledge, this report is the first to document extensive neuronophagia and microglial nodules in the inferior olives and dentate nuclei without pronounced inflammatory infiltrates in a confirmed SARS-CoV-2 autopsy.

## Data Availability

Not applicable.
